# Up-regulation of neural and cell cycle-related microRNAs in brain of amyotrophic lateral sclerosis mice at late disease stage

**DOI:** 10.1186/s13041-015-0095-0

**Published:** 2015-01-28

**Authors:** Stefania Marcuzzo, Silvia Bonanno, Dimos Kapetis, Claudia Barzago, Paola Cavalcante, Sara D’Alessandro, Renato Mantegazza, Pia Bernasconi

**Affiliations:** Neurology IV - Neuromuscular Diseases and Neuroimmunology Unit, Fondazione Istituto Neurologico “Carlo Besta”, Via Celoria 11, Milan, 20133 Italy

**Keywords:** G93A-SOD1 mice, microRNAs, Neural stem progenitor cells

## Abstract

**Background:**

Amyotrophic lateral sclerosis (ALS) is a fatal neurodegenerative disease characterized by selective motor neuron degeneration in motor cortex, brainstem and spinal cord. microRNAs (miRNAs) are small non-coding RNAs that bind complementary target sequences and modulate gene expression; they are key molecules for establishing a neuronal phenotype, and in neurodegeneration. Here we investigated neural miR-9, miR-124a, miR-125b, miR-219, miR-134, and cell cycle-related miR-19a and -19b, in G93A-SOD1 mouse brain in pre-symptomatic and late stage disease.

**Results:**

Expression of miR-9, miR-124a, miR-19a and -19b was significantly increased in G93A-SOD1 whole brain at late stage disease compared to B6.SJL and Wt-SOD1 control brains. These miRNAs were then analyzed in manually dissected SVZ, hippocampus, primary motor cortex and brainstem motor nuclei in 18-week-old ALS mice compared to same age controls. In SVZ and hippocampus miR-124a was up-regulated, miR-219 was down-regulated, and numbers of neural stem progenitor cells (NSPCs) were significantly increased. In G93A-SOD1 brainstem motor nuclei and primary motor cortex, miR-9 and miR-124a were significantly up-regulated, miR-125b expression was also increased. miR-19a and -19b were up-regulated in primary motor cortex and hippocampus, respectively. Expression analysis of predicted miRNA targets identified miRNA/target gene pairs differentially expressed in G93A-SOD1 brain regions compared to controls.

**Conclusions:**

Hierarchical clustering analysis, identifying two clusters of miRNA/target genes, one characterizing brainstem motor nuclei and primary motor cortex, the other hippocampus and SVZ, suggests that altered expression of neural and cell cycle-related miRNAs in these brain regions might contribute to ALS pathogenesis in G93A-SOD1 mice. Re-establishing their expression to normal levels could be a new therapeutic approach to ALS.

**Electronic supplementary material:**

The online version of this article (doi:10.1186/s13041-015-0095-0) contains supplementary material, which is available to authorized users.

## Background

Amyotrophic lateral sclerosis (ALS) is a fatal neurodegenerative disease characterized by selective motor neuron degeneration, which leads to atrophy of the associated muscles [1]. Ten percent of ALS patients have genetically determined disease caused by mutations in a heterogeneous set of genes [1]. Despite increasing knowledge of pathogenetic mechanisms, no effective treatments are available for ALS, highlighting the need to identify additional mechanisms that can serve as therapeutic targets.

The G93A-SOD1 transgenic mouse model of ALS, which overexpresses the G93A mutated human SOD1 gene, shows symptoms and neuropathological features similar to those of human ALS [2]. By magnetic resonance imaging (MRI) we detected skeletal muscle atrophy in G93A-SOD1 mice at a very early stage, in the absence of motor symptoms or neurodegenerative changes in brainstem motor nuclei, suggesting that neural deficits develop well before the disease becomes clinically evident [3].

Proliferation and differentiation of neural stem progenitor cells (NSPCs) have been found altered in various neurodegenerative conditions [4,5]. In ALS mice, as the disease progresses, NSPCs attempt to proliferate in the subventricular zone (SVZ) [6] and subgranular zone (SGZ) [7] – the two largest neurogenic areas of the central nervous system [8,9] – and also in motor cortex [6]. A significant increase in NSPC number has also been reported in brainstem motor nuclei of G93A-SOD1 mice [10], in association with selective neuronal degeneration as revealed by MRI [11]. Proliferating NSPCs and neuroblasts have also been identified in the SVZ of an ALS patient with frontotemporal dementia, suggesting that neural proliferation takes place in response to the disease [12].

MicroRNAs (miRNAs) are small non-coding RNAs that bind complementary target sequences to modulate gene expression; they are key molecules controlling cell proliferation, differentiation and neurogenesis [13-19]. In particular they are known to be involved both in establishing the neuronal phenotype and in neurodegeneration [20-26]. In fact miRNAs have been found either up-regulated or down-regulated in ALS spinal cord, muscle tissue and peripheral blood mononuclear cells (reviewed in ref. [27]).

We recently found that the expression of neural (miR-9, miR-124a) and cell cycle-related (miR-19a and -19b) miRNAs was significantly associated with altered neuronal fate of cultured ependymal stem/progenitor cells isolated from spinal cord of ALS mice, and that these alterations became more marked as disease progressed [28], suggesting that these miRNAs are involved in ALS pathogenesis and progression.

Taking these spinal cord findings as our starting point, in the present study we investigated whether disease progression in G93A-SOD1 mice was associated with altered expression of miRNAs in various brain regions. We investigated miR-9, miR-124a, miR-19a and -19b, as in our previous study [28]. We also investigated miR-125 and miR-219, implicated in astrocyte and oligodendrocyte regulation [29,30], since the ALS brain is known to be characterized by neurodegeneration and astrogliosis. Finally, we investigated miR-134, implicated in neuronal morphogenesis and synaptic plasticity [31].

In the present study, we first investigated the expression of miR-9, miR-124a, miR-19a, miR-19b and miR-134 in the whole brain of G93A-SOD1 mice in comparison with that of B6.SJL and Wt-SOD1 control mice, in asymptomatic (week 8) and late stage disease (week 18). We found that the expression of miR-134 did not differ significantly between ALS and control brain, while the expression of the other miRNAs did. We next analyzed the expression of miR-9, miR-124a, miR-19a and -19b, miR-125 and miR-219 in manually dissected SVZ, hippocampus, primary motor cortex and brainstem motor nuclei in 18-week-old ALS mice compared to same age controls. We found that the expression of all the miRNAs investigated was significantly altered in several of these brain areas.

Our findings show that the expression of miRNAs regulating neural and cell cycle processes is altered in G93A-SOD1 mouse brain in late stage disease. We propose that normalization of altered miRNA expression might be a useful therapeutic approach to ALS.

## Results

### miRNA expression in G93A-SOD1 whole brain in relation to disease progression

**Figure 1:**
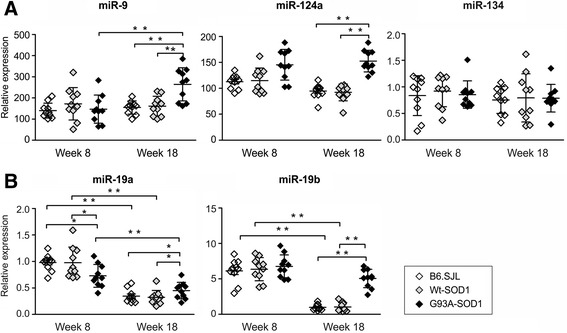
**Neural and cell cycle**-**related miRNAs are altered in G93A-SOD1 mouse brain as disease progresses.** RT-PCR analysis of brain-specific **(A)** and cell cycle-related **(B)** miRNAs in total RNA extracted from whole brain of G93A-SOD1, B6.SJL and Wt-SOD1 mice, at postnatal weeks 8 and 18 (ten mice per group). Each point represents a single brain. Relative expression data are presented as means ± SD. *p < 0.05; **p < 0.01; limma moderated t-test.

At 8 weeks expression levels of miR-9, miR-124a, miR-19a and -19b did not differ significantly between whole brains of G93A-SOD1, B6.SJL and Wt-SOD1 mice, except for miR-19a, which was significantly down-regulated in ALS brain (*p* < 0.05) (Figure 1). At week 18, miR-9, miR-124a, miR-19a and -19b levels were significantly higher in G93A-SOD1 than control brains (*p* < 0.01 both B6.SJL and Wt-SOD1) (Figure 1). In addition, miR-9 was significantly up-regulated in G93A-SOD1 brain compared to week 8 (*p* < 0.01).

As is evident in Figure 1, expression levels of the selected miRNAs did not differ between Wt-SOD1 and B6.SJL controls, indicating that expression of the human wild-type SOD1 transgene does not affect the miRNA expression in these mice, and that Wt-SOD1 mice are suitable controls for this study.

Interestingly, at week 18, expression levels of miR-9 and miR-124a, but not miR-134, were significant lower in G93A-SOD1 whole spinal cord than in Wt-SOD1 spinal cord (*p* < 0.05 and *p* < 0.01, respectively), whereas miR-19a and -19b expression levels were significantly higher in ALS than Wt-SOD1 mice (*p* < 0.01 and *p* < 0.05, respectively) (Additional file 1: Figure S1).

### miRNA expression in selected brain regions

**Figure 2:**
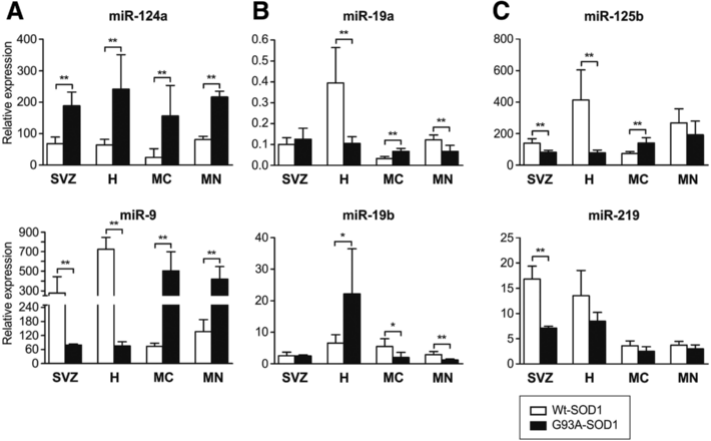
**Brain-specific, cell cycle- and glia-related miRNA expression is altered compared to control in different regions of G93A-SOD1 brain.** RT-PCR analysis of brain-specific **(A)**, cell cycle- **(B)** and glia- **(C)** related miRNAs in subventricular zone (SVZ), hippocampus (H), primary motor cortex (MC), and brainstem motor nuclei (MN) of G93A-SOD1 and Wt-SOD1 brain at late stage disease. Data are means of relative expression ± SD of 5 mice per group. *p < 0.05; **p < 0.01; limma moderated t-test.

We next wondered whether altered miRNA expression in the brain of 18-week-old ALS mice would be evident in specific brain regions concerned with neurogenesis (SVZ and hippocampus) and affected by ALS (primary motor cortex and brainstem motor nuclei). We also analyzed miR-125b, marker of reactive astrocytes [29], and miR-219, marker of oligodendrocyte differentiation [30], to investigate glial cell involvement in these brain regions. We found that miR-124a expression was significantly greater in SVZ, hippocampus, primary motor cortex, and brainstem motor nuclei of ALS than Wt-SOD1 mice (*p* < 0.01). miR-9 expression was lower in SVZ and hippocampus, and significantly greater in primary motor cortex and brainstem motor nuclei (*p* < 0.01) in ALS compared to control (Figure 2A). miR-19a was significantly down-regulated in hippocampus and brainstem motor nuclei and significantly up-regulated in primary motor cortex of G93A-SOD1 compared to control (*p* < 0.01). miR-19b was up-regulated in hippocampus (*p* < 0.05) and down-regulated in primary motor cortex and brainstem motor nuclei (*p* < 0.05 and *p* < 0.01, respectively) (Figure 2B). miR-125b was significantly down-regulated in G93A-SOD1 SVZ and hippocampus (*p* < 0.01) and significantly up-regulated in primary motor cortex (*p* < 0.01). Finally, miR-219 was significantly lower in ALS SVZ than control SVZ (*p* < 0.01) (Figure 2C). miR-125b and miR-219 tended to be expressed at higher levels (not significant) in cervical, thoracic and lumbar spinal cord of ALS than control mice (Additional file 2: Figure S2).

### Expression of miRNA targets

**Figure 3:**
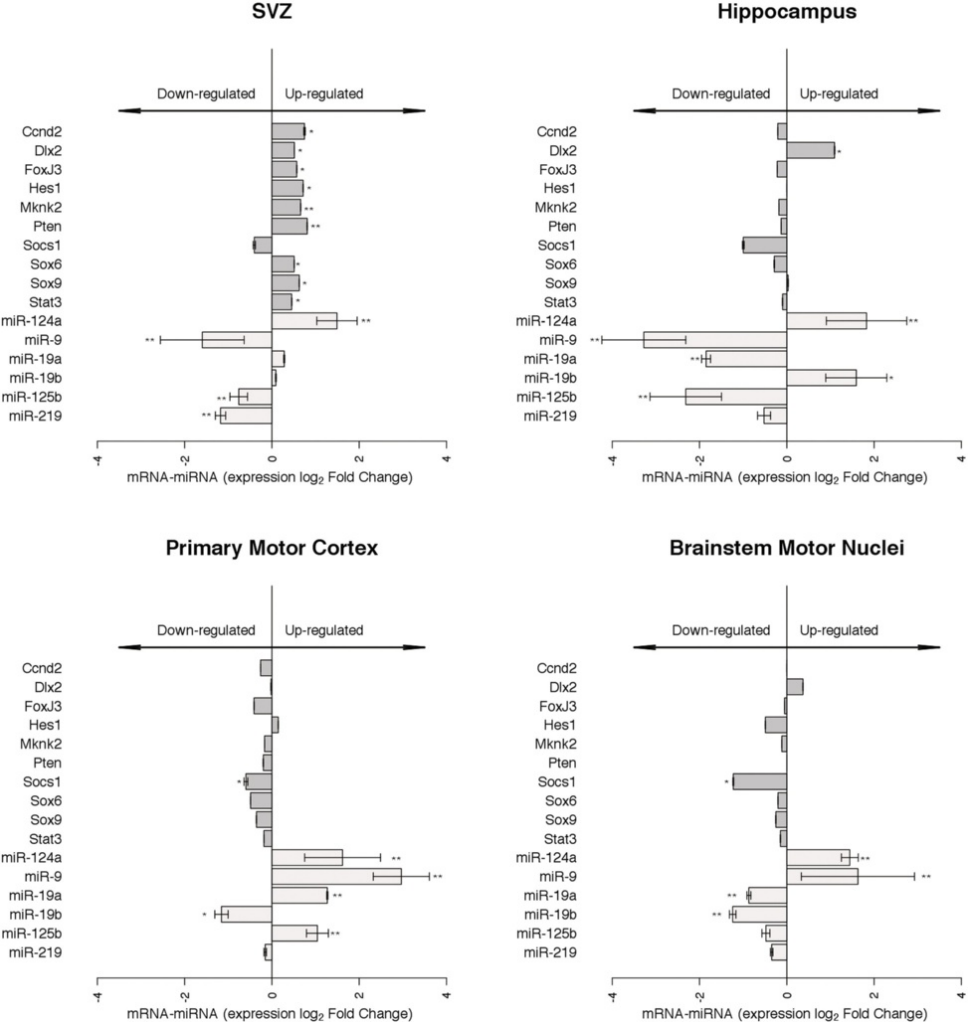
**Altered expression of predicted miRNA targets in distinct regions of G93A-SOD1 mouse brain.** Data are presented as means ± SD of log2 of fold changes of 2^-ΔCT^ expression in G93A-SOD1 relative to Wt-SOD1 (grey bars) at late stage disease (week 18). Significant changes in mRNA (*p < 0.05, **p < 0.01, grey bars) and miRNA (*p < 0.05, **p < 0.01, light grey bars) expression relative to control are indicated. Limma moderated t-test.

Based on miRWalk database prediction [32] and on the literature [28,30,33-37], the following miRNA target mRNAs were identified: cyclin D2 (Ccnd2), Dlx2, forkhead box protein J3 (FoxJ3), hairy and enhancer of split 1 (Hes1), Jagged 1 (Jag1), MAP kinase interacting serine/threonine kinase 2 (Mknk2), nuclear receptor subfamily 2 (Nr2e1), phosphatase and tensin homolog (Pten), specific E3 ubiquitin protein ligase 1 (Smurf1), suppressor of cytokine signaling 1 (Socs1), sex determining region Y(SRY)-box 6 (Sox6), Sox9 and signal transducer and activator of transcription 3 (STAT3). Their expression levels were quantified in 18-week-old ALS and control brain. Jag1, Nr2e1, and Smurf1 mRNA levels did not differ significantly between ALS and controls in any brain area (data not shown). In SVZ, all other mRNAs, except Socs1, were significantly more expressed in ALS than control brain (*p* < 0.05 and *p* < 0.01, Figure 3). In hippocampus, primary motor cortex and brainstem motor nuclei, the mRNAs of most putative target genes were non-significantly reduced in ALS compared to control; exceptions were a significant increase of Dlx2 in hippocampus, and a significant decrease of Socs1 in primary motor cortex and brainstem motor nuclei (*p* < 0.05, Figure 3).

### Correlations between miRNA and mRNA expression in 18-week-old ALS mouse brain

Pearson’s correlation analysis was applied to each miRNA and its putative target, to provide evidence of relationships between them, in brain regions of 18-week-old ALS mice. Since miRNAs mainly act as negative regulators, we focused on inverse correlations, considering coefficients (r) less than −0.5 as indicating a good inverse correlation. The results of the analysis are reported in Table 1. The most conspicuous correlations were found in areas (primary motor cortex and brainstem motor nuclei) characterized by marked motor neuron loss.

**Table 1:** **List of gene targets for each miRNA and inverse correlations in each brain region investigated of 18-week old G93A-SOD1 mice**

**miRNA**	**Gene target**	**SVZ**	**Hippocampus**	**Primary motor cortex**	**Brainstem motor nuclei**
miR-124a	- Distal-less homeobox 2 (Dlx2)	r > − 0.5	r > − 0.5	**r = − 0.96**	**r = − 0.96**
	- Jagged (Jag1)	r > − 0.5	r > − 0.5	r > − 0.5	r > − 0.5
	- Sex determining region Y(SRY)-box 9 (Sox9)	r > − 0.5	r > − 0.5	r > − 0.5	**r = − 0.51**
	- Signal transducer and activator of transcription 3 (STAT3)	r > − 0.5	r > − 0.5	r > − 0.5	**r = − 0.89**
miR-9	- Hairy and enhancer of split (Hes1)	r > − 0.5	r > − 0.5	r > − 0.5	r > − 0.5
	- Nuclear receptor subfamily (Nr2e1)	r > − 0.5	r > − 0.5	r > − 0.5	r > − 0.5
	- Phoshatase and tensin homolog (Pten)	r > − 0.5	r > − 0.5	r > − 0.5	**r = − 0.54**
	- STAT3	**r = − 0.77**	**r = − 0.95**	r > − 0.5	**r = − 0.51**
miR-19a	- Cyclin D2 (Ccnd2)	r > − 0.5	**r = − 0.76**	r > − 0.5	r > − 0.5
	- Pten	r > − 0.5	r > − 0.5	r > − 0.5	r > − 0.5
	- Suppressor of cytokine signalling 1 (Socs1)	r > − 0.5	**r = − 0.88**	r > − 0.5	r > − 0.5
	- Sex determining region Y(SRY)-box 6 (Sox6)	**r = − 0.96**	r > − 0.5	**r = − 0.96**	r > − 0.5
miR-19b	- Ccnd2	r > − 0.5	r > − 0.5	r > − 0.5	r > − 0.5
	- Pten	**r = − 0.85**	r > − 0.5	r > − 0.5	r > − 0.5
	- Socs1	**r = − 0.90**	r > − 0.5	r > − 0.5	r > − 0.5
	- Sox6	**r = − 0.83**	r > − 0.5	**r = − 0.96**	r > − 0.5
miR-125b	- Forkhead box protein J3 (FoxJ3)	r > − 0.5	r > − 0.5	r > − 0.5	r > − 0.5
	- Sox6	r > − 0.5	r > − 0.5	**r = − 0.96**	r > − 0.5
miR-219	- FoxJ3	r > − 0.5	r > − 0.5	**r = − 0.96**	r > − 0.5
	- MAP kinase interacting serine/threonine kinase 2 (Mknk2)	r > − 0.5	r > − 0.5	**r = − 0.99**	r > − 0.5
	- Pten	r > − 0.5	r > − 0.5	**r = − 0.50**	r > − 0.5
	- Specific E3 ubiquitin protein ligase 1 (Smurf1)	r > − 0.5	r > − 0.5	r > − 0.5	r > − 0.5

miRNA targets were predicted from published data and in-silico modeling using miRWalk database and default score parameters (http://www.umm.uni-heidelberg.de/apps/zmf/mirwalk/). Pearson’s correlation coefficients (r) were used to corroborate relationships between miRNAs and target mRNA expression levels in G93A-SOD1 mouse brain regions at week 18. Coefficients less than −0.5 (bold) were considered to indicate a good inverse correlation.

### NSPCs in SVZ and hippocampus of ALS mice

**Figure 4:**
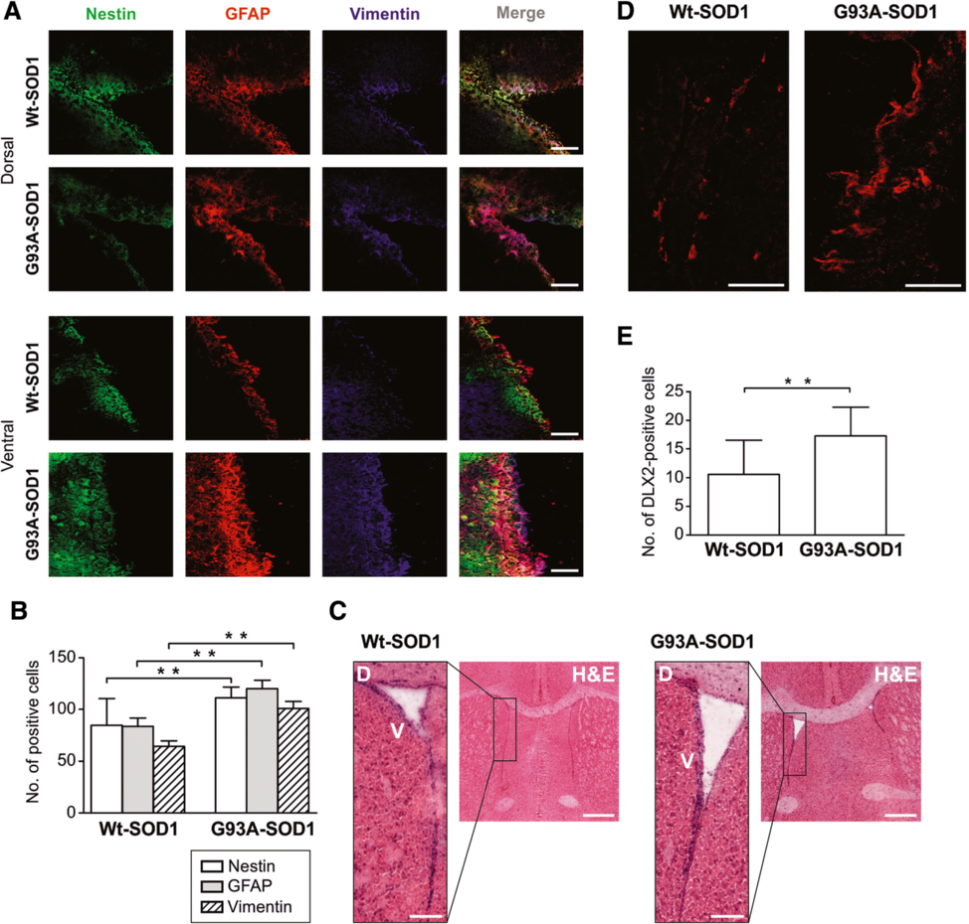
**Increased numbers of differentiating NSPCs in SVZ of G93A-SOD1 brain. (A)** Confocal microscopy images of dorsal and ventral regions of SVZ in G93A-SOD1 and Wt-SOD1 brain at postnatal week 18, stained for nestin (green), GFAP (red), and vimentin (blue). Scale bar = 50 μm. **(B)** Quantification of nestin-, GFAP-, and vimentin-positive cells in SVZ of G93A-SOD1 and Wt-SOD1 mice. Data are means ± SD of 3 mice per group. **p < 0.001; limma moderated t-test. **(C)** Hematoxylin and eosin staining of SVZ sections adjacent to those analyzed by confocal microscopy. Scale bar = 50 μm. D: dorsal. V: ventral. **(D)** Confocal microscopy images showing Dlx2-stained cells in ventral SVZ in G93A-SOD1 and Wt-SOD1 brain. Scale bar = 50 μm. **(E)** Quantification of Dlx2-positive cells in G93A-SOD1 and Wt-SOD1 SVZ. Data are means ± SD of 3 mice per group. **p < 0.001; limma moderated t-test.

**Figure 5:**
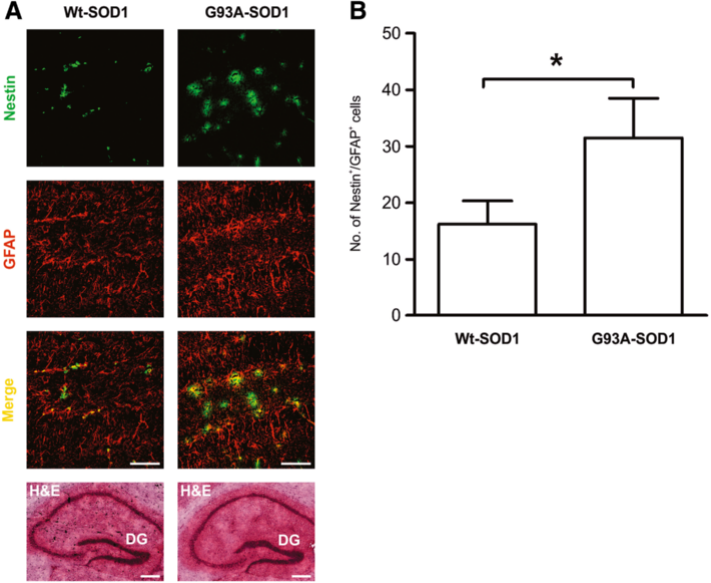
**Increased numbers of NSPCs in hippocampus of G93A-SOD1 mouse at postnatal week 18. (A)** Confocal microscopy images of hippocampus dentate gyrus (DG) in G93A-SOD1 and Wt-SOD1 mouse, stained for nestin (green) and GFAP (red). Scale bar = 50 μm. Hematoxylin and eosin staining of hippocampus (lower panel) sections adjacent to those analyzed by confocal microscopy. Scale bar = 50 μm. **(B)** Quantification of nestin- and GFAP-positive cells in G93A-SOD1 and Wt-SOD1 brain. Data are means ± SD of 3 mice per group. *p < 0.05; limma moderated t-test.

The SVZ is the primary source of new neurons in adult brain. Neurons arise from unipolar bipolar type B stem cells that express nestin and vimentin and/or glial fibrillary acidic protein (GFAP): they give rise to transit-amplifying type C cells, and in turn produce type A neuroblasts expressing Dlx2 – which are migratory and proliferative neuronal precursors [8]. Immunohistochemical analysis for nestin, GFAP and vimentin revealed significantly more unipolar and bipolar cells positive for these markers in the dorsal and ventral SVZ of 18-week-old ALS mice compared with same areas in Wt-SOD1 mice (*p* < 0.001; Figure 4A-C). We also found significantly more Dlx2-positive cells in the ventral SVZ of ALS than Wt-SOD1 mice (*p* < 0.001; Figure 4D, E). These findings suggest that neurogenesis is occurring in the SVZ of ALS mice at late stage disease. In the adult mammalian brain neurogenesis also occurs in the hippocampal dentate gyrus [38]. Immunohistochemical analysis of hippocampal dentate gyrus revealed significantly more nestin- and GFAP-positive NSPCs in 18-week-old ALS mice than same-age controls (*p* < 0.05; Figure 5).

### Heat map of miRNAs and predicted gene targets in regions of G93A-SOD1 brain

**Figure 6:**
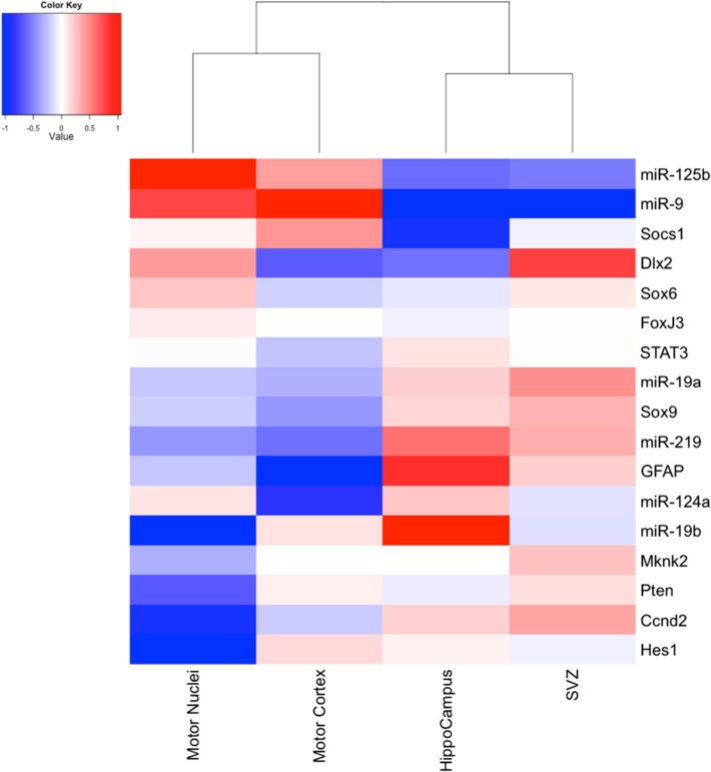
**Heat map of miRNAs and predicted gene targets in regions of G93A-SOD1 mouse brain.** Regions investigated were brainstem motor nuclei, primary motor cortex, hippocampus and SVZ at week 18. Expression data are means of log_2_ fold changes (relative to mean values for all brain areas investigated). Blue indicates down-regulation and red indicates up-regulation of miRNAs and predicted gene targets, respectively. Hierarchical clustering analysis identifies two clusters of miRNAs and their predicted targets: one characterizing brainstem motor nuclei and primary motor cortex (areas of neurodegeneration); the other characterizing hippocampus and SVZ (areas of neurogenesis).

Figure 6 shows expression levels of miRNAs and target mRNAs relative to mean values in all brain areas investigated. Hierarchical clustering analysis identified two clusters of miRNAs and their predicted targets: one characterizing brainstem motor nuclei and primary motor cortex; the other characterizing hippocampus and SVZ.

## Discussion

In the present study we have found that in whole brain and brain regions concerned with neurogenesis (SVZ and hippocampus) and affected by motor neuron degeneration (primary motor cortex and brainstem motor nuclei), altered expression of neural fate miR-124a and miR-9 (but not miR-134), cell cycle-related miR-19a and -19b, astrocyte-related miR-125b and oligodendrocyte -related miR-219 occur in late stage disease (18 weeks).

miR-124a, highly conserved during evolution, is one of the most highly expressed miRNAs in the CNS [15,39]. In mouse it plays a key role in promoting neuronal differentiation of NSPCs to mature neurons by inhibiting non-neuronal genes such as Sox9 [15]. We found miR-124a up-regulation in SVZ, hippocampus, primary motor cortex and brainstem motor nuclei of 18-week-old G93A mice probably reflecting active neuronal differentiation in response to motor neuron degeneration [5,6,40]. NSPCs are normally quiescent in the brain, but under pathological conditions switch to active in neurogenic regions [41]. NSPCs can generate Dlx2-expressing neuroblasts in SVZ [8,15]. It is noteworthy that we found significantly more Dlx2-positive neuroblasts in ALS SVZ than control. We also found a good inverse correlation between miR-124a and Sox9 expression in brainstem motor nuclei – a region markedly affected by motor neuron loss in ALS, as also found in our MRI study on late stage disease ALS mice [3].

miR-9 – an evolutionarily-conserved brain-enriched miRNA [39] – might play an important role in ALS neurodegeneration. We found significantly increased expression of miR-9 in primary motor cortex and brainstem motor nuclei, and significantly down-regulated miR-9 in SVZ and hippocampus. Our finding that miR-124a and miR-9 are up-regulated in primary motor cortex and brainstem motor nuclei therefore suggests a compensatory response to motor neuron degeneration in these areas highly affected by the disease process.

Both miR-124a and miR-9 target STAT3 [42]. In developing CNS, STAT3 activation reduces neurogenesis from neural stem cells [43] in favor of differentiation to astrocytes [44]. We found a good negative correlation between miR-124a and miR-9 and STAT3 in brainstem motor nuclei, again suggesting that neural precursors are being directed toward a neuronal rather than glial fate in this damaged area. However in brainstem STAT3 reduction was not significant. miR-9 also correlated negatively with STAT3 in SVZ and hippocampus: STAT3 levels were slightly increased in these regions while miR-9 was significantly down-regulated, again suggesting compromised neurogenesis in these neurogenic areas.

The later finding is interesting, since miR-9’s absence in mice causes the premature birth of cortical neurons and suppression of neural precursor proliferation in the ventricular and subventricular zones [45] and this might compromise neuroregeneration in the ALS mouse.

In our previous study on cultured ependymal stem/progenitor cells isolated from G93A-SOD1 mouse spinal cord at week 18, miR-9 correlated negatively with STAT3 in neural differentiated cells [28], again suggesting involvement of this miRNA-mRNA pair in the regulation of stem cell signaling pathways.

With regard to miR-19a and -19b, these are involved in cell cycle regulation and their expression is enhanced in several stem cell types [13,46]. We found that miR-19a was significantly down regulated in hippocampus, while, as expected, Ccnd2 levels correlated inversely with miR-19a. Ccnd2 is known to enhance proliferation [47]. We there conjecture Ccnd2 activity is tending to enhance proliferation in this brain area, as also suggested by our finding of increased number of NSPCs in this brain region.

We found that miR-19b was down-regulated in motor cortex and the levels of predicted target Sox6 correlated negatively with miR-19b in this brain area. Sox6 is known to promote the survival and renewal of neural precursor cells and is enhancing the survival of neural precursors in this brain area [48].

As regards miR-125b, which is expressed in neurons and astrocytes and seems to be involved in the astrogliosis that occurs in Alzheimer disease [49], we found that this miRNA was significantly down-regulated in SVZ and hippocampus – areas in which differentiating NSPCs were significantly increased in G93A-SOD1 mice compared to control – but significantly up*-*regulated in primary motor cortex in association with reduced levels of Sox6. These data suggest that miR-125b overexpression in primary motor cortex and also spinal cord (Additional file 2: Figure S2) is functionally associated with the corticospinal tract degeneration characteristic of ALS.

Our finding of significantly decreased miR-219 expression in SVZ suggests an imbalance between oligodendrocyte and neuronal precursors during NSPC differentiation in that brain region, as further supported by the increased numbers of Dlx2-positive neuroblasts found there.

When we applied hierarchical clustering to the expression of miRNAs and their putative mRNA targets, two clusters emerged: one characterizing brainstem motor nuclei and primary motor cortex; the other characterizing hippocampus and SVZ (Figure 6). This finding is noteworthy since primary motor cortex and brainstem motor nuclei are the regions rich in upper and lower motor neuron cell bodies, whereas SVZ and hippocampus are neurogenic areas where (unsuccessful) neurogenesis in response to motor neuron degeneration seems to be occurring.

In the present study we focused on expression levels of mature miRNAs; however studies have shown that various regulatory factors are involved in miRNA biogenesis (reviewed in ref. [27]) and that miRNA expression is tightly regulated by mechanisms that influence transcription, processing and turnover (reviewed in ref. [27,50]). Several gene-products associated with ALS have been also shown to be involved in miRNA pathways. For example, TAR DNA-binding protein TDP-43 – component of Dicer and Drosha complexes and important for miRNA biogenesis – selectively regulates pri-miRNA processing by binding to primary transcripts of specific miRNAs. FUS/translocated in liposarcoma protein also binds pre-mRNA molecules. Both TDP-43 and FUS/TLS influence the fate of miRNAs by regulating splicing, transport, stability, and translation (reviewed in ref. [27]). These findings suggest that mutations in ALS-related genes can lead to altered miRNA biogenesis and function, which may in turn contribute to the ALS phenotype. We hope to present the results of our ongoing studies on alterations in miRNA biogenesis pathways in the mouse ALS model in a subsequent publication.

## Conclusions

The aberrant expression of miRNAs in many human diseases [51] makes them potential drug targets, and artificial modulation of dysregulated endogenous miRNAs, through use of antagonists or mimics, is being increasingly considered for therapy [52-55]. We have identified miRNAs, which appear to be involved in the pathogenesis of ALS in mice: these should be further investigated to develop therapies that can restore normal miRNA levels and hopefully ameliorate the course of the disease.

## Methods

Reagents, source companies and working dilutions are listed in Table 2.

**Table 2:** **Reagents, source companies and working dilutions**

**Source**			
Animals	G93A-SOD1 (B6SJL-Tg(SOD1*G93A)1Gur)	Charles River (Wilmington, MA)	
	Wild-type (Wt)-SOD1 (B6SJLTg(SOD1)2Gur/J)	Charles River (Wilmington, MA)	
	B6.SJL	Charles River ((Wilmington, MA)	
			Working dilution
Primary	-Rabbit anti-mouse glial fibrillary acidic protein		
Antibodies	(GFAP) IgG antibody	Dako Cytomation (Glostrup, Denmark)	1:300
	-Chicken anti-mouse vimentin IgG antibody	Novus Biological	1:500
	-Mouse anti-mouse nestin IgG antibody	Millipore (Billerica, MA)	1:200
	-Goat anti-mouse Dlx2 IgG antibody	Santa Cruz (Heidelberg, Germany)	1:50
Secondary	-Cy3-conjugated goat anti-rabbit IgG	Jackson ImmunoResearch (Newmarket, UK)	1:600
antibodies	-AMCA-conjugated anti-chicken IgG	Jackson ImmunoResearch (Newmarket, UK)	1:100
	-Cy2-conjugated goat anti-mouse IgG	Jackson ImmunoResearch (Newmarket, UK)	1:200
	-Cy2-conjugated donkey anti-goat IgG	Jackson ImmunoResearch (Newmarket, UK)	1:200
	-Isotype-specific non-immune IgG (control)	Dako Cytomation (Glostrup, Denmark)	1:200
	-Normal goat serum (control)	Vector Laboratories (Peterborough, UK)	
Reagents for	-Optical Cutting Temperature Compound	Bio-Optica (Milan, Italy)	
sample conservation			
Microscopes	-Aperio scanner	Nikon (GMBH, Germany)	
	-Aperio Image Scope v12.0.0.5039	Nikon (GMBH, Germany)	
	-Eclipse TE-2000-E	Nikon (Tokyo, Japan)	
Reagents	-Trizol	Life Technologies (Foster City, MA)	
qReal-Time PCR	-2100Nano Bioanalyzer	Agilent Technologies (Waldbronn, Germany)	
	-TaqMan MicroRNA reverse Transcription Kit	Life Technologies (Foster City, MA)	
	-miRBase ID mmu-miR-9-5p	Life Technologies (Foster City, MA)	
	-miRBase ID mmu-miR-124-3p	Life Technologies (Foster City, MA)	
	-miRBase ID mmu-miR-134-5p	Life Technologies (Foster City, MA)	
	-miRBase ID mmu-miR-125b-5p	Life Technologies (Foster City, MA)	
	-miRBase ID mmu-miR-219a-5p	Life Technologies (Foster City, MA)	
	-miRBase ID mmu-miR-19a-3p	Life Technologies (Foster City, MA)	
	-miRBase ID mmu-miR-19b-3p	Life Technologies (Foster City, MA)	
	-miRBase ID xtr-miR-24a-3p	Life Technologies (Foster City, MA)	
Reagents for			
miRNA	-SuperScript Vilo cDNA Synthesis kit	Life Technologies (Foster City, MA)	
target validation	-Universal PCR master mix	Life Technologies (Foster City, MA)	
	-Taqman Array Fast Plate	Life Technologies (Foster City, MA)	
	-Mm-Ccnd2-Assay ID00438070_m1	Life Technologies (Foster City, MA)	
	-Mm-Dlx2-Assay ID00438427_m1	Life Technologies (Foster City, MA)	
	-Mm-Foxj3-Assay ID00554610_m1	Life Technologies (Foster City, MA)	
	-Mm-Hes1-Assay ID01342805_m1	Life Technologies (Foster City, MA)	
	-Mm-Jag1-Assay ID00496902_m1	Life Technologies (Foster City, MA)	
	-Mm-Mknk2- Assay ID00458026_m1	Life Technologies (Foster City, MA)	
	-Mm-Nr2e1-Assay ID00438427_m1	Life Technologies (Foster City, MA)	
	-Mm-Pten-Assay ID00477208_m1	Life Technologies (Foster City, MA)	
	-Mm-Smurf1-Assay ID00547102_m1	Life Technologies (Foster City, MA)	
	-Mm-Socs1-Assay ID01219775_g1	Life Technologies (Foster City, MA)	
	-Mm-Sox6-Assay ID00488393_m1	Life Technologies (Foster City, MA)	
	-Mm-Sox9-Assay ID00448840_m1	Life Technologies (Foster City, MA)	
	-Mm-Stat3-Assay ID01219775_m1	Life Technologies (Foster City, MA)	
	-Mm-GFAP-Assay ID0125033_m1	Life Technologies (Foster City, MA)	
	-Mm-18 s-Assay ID03928990_g1	Life Technologies (Foster City, MA)	

### Animals

Transgenic G93A-SOD1 (B6SJL-Tg(SOD1*G93A)1Gur), wild-type (Wt)-SOD1 (B6SJLTg(SOD1)2Gur/J) and B6.SJL mice were maintained and bred at the animal house of the C Besta Neurological Institute, in compliance with institutional guidelines and international law (EEC Council Directive 86/609, OJL 358, 1, December 12, 1987, NIH Guide for the Care and Use of Laboratory Animals, U.S. National Research Council, 1996). G93A- and Wt-SOD1 progeny were identified by RT-PCR of the human SOD1 gene [3]. Males were used in all experiments. They were killed by exposure to CO_2_ at week 8 (pre-symptomatic) or week 18 (late stage disease) [3].

### Isolation of specific brain and spinal cord regions

Each brain region was identified with the aid of the coordinates shown in the mouse brain atlas [56]. The region containing brainstem motor nuclei (including facial, ambiguous, trigeminal and hypoglossal nuclei) was also recognized and isolated with the aid of MR images, as described in a previous paper [3]. We manually dissected out the brain regions of interest using a dissecting microscope and scalpel, as described by Matsumura et al. [38]. We initially removed the olfactory bulbs with a coronal cut about 2 mm from the rostral surface of the frontal lobes. We then cut a 3 mm-thick slice containing the primary motor cortex, secondary motor cortex and dorsal and ventral parts of the subventricular zone (SVZ). In this slice, we identified the corpus callosum as separating the motor cortex (above) from the SVZ (below). The SVZ was also identified as forming the wall of the lateral ventricles. A 2 mm-thick slice was taken next which contained the hippocampus, readily identified by visual inspection, which was carefully dissected out. A successive coronal section of about 2 mm thickness was taken, which clearly showed the cerebral aqueduct. Around and underlying this were the brainstem motor nuclei and the visually evident trigeminal nucleus. This area was separated out using a scalpel. A final 1 mm-thick slice was taken. This contained the evident fourth ventricle; below this continued the area of the brainstem motor nuclei, which was also separated out using a scalpel and united with the material of the previous section.

Total spinal cord was dissected out and cut into cervical, thoracic, and lumbar sections. The cervical section comprised the part from the top of the spinal cord to the cervical enlargement. The thoracic segment was that from the cervical enlargement to the last rib. The remaining spinal cord was the lumbar segment.

### Quantitative real-time PCR to determine miRNAs

Total RNA was extracted with TRIzol reagent from: whole brain (300–400 mg); spinal cord (100–200 mg); SVZ, hippocampus, primary motor cortex and brainstem motor nuclei and also cervical, thoracic and lumbar spinal cord. The RNA was retrotranscribed to cDNA using TaqMan MicroRNA Reverse Transcription Kits with primers specific for miR-9, miR-19a, miR-19b, miR-124a, miR-125b, miR-134, miR-219 and miR-24. miR-24, used as endogenous control [57] was stably expressed in whole brain and the brain regions studied of G93A-SOD1, B6.SJL and Wt-SOD1 mice (as shown by standard deviation of Ct values < 0.5). cDNA aliquots corresponding to 15 ng total RNA were amplified by quantitative real time PCR in triplicate, with Universal PCR master mix and specific pre-designed TaqMan MicroRNA assays. miRNA levels were normalized to miR-24 and expressed as fold changes using the formula 2^-ΔCt^.

### Prediction of miRNA gene targets

miRNA targets were predicted in-silico using the miRWalk database using the default score parameters [32]. We also identified potentially dysregulated target genes from the literature and our previous studies [28,30,33-37]. We chose to include those genes strictly implicated in cell-cycle regulation and cell function signaling neurogenesis. The mRNA targets of each miRNA are listed in Table 1 and the prediction tool applied to identify them is reported in the footnote of Table 1.

### mRNA real-time PCR

Total RNA, previously examined for miRNA expression in distinct brain regions, was retrotranscribed using SuperScript Vilo cDNA Synthesis kit. cDNA (20 ng total RNA) was amplified by qPCR in duplicate, using Universal PCR master mix and Taqman Array Fast Plate, assembled with primer and probe sets for the identified 13 miRNA target genes and one housekeeping gene, 18s [58]. 18s was stably expressed in distinct brain regions of G93A-SOD1 and WT-SOD1 mice; standard deviation of Ct values: < 0.5. In addition, GFAP mRNA expression levels were measured as marker for astrocytes.

### Immunostaining

Brain tissue was embedded in optimal cutting temperature compound immediately upon removal and stored at −80°C pending histological analyses. Cryostat 15 μm-thick sections were cut in correspondence to SVZ and hippocampus regions, stained with hematoxylin-eosin, and images were digitally acquired with the Aperio ScanScope system. Adjacent sections were fixed in 4% paraformaldehyde, treated with 0.1% Triton X-100 for 10 min, and washed with PBS. Sections were then incubated with 10% normal goat serum or bovine serum albumin and with the following primary antibodies, overnight at 4°C: GFAP, marker for astrocytes; vimentin, for glial precursors; nestin, for NSPCs; distal-less homeobox 2 (Dlx2), for neuronal precursors. After washing, samples were incubated for 1 hour at room temperature with secondary antibodies (Cy3-conjugated goat anti-rabbit IgG, AMCA-conjugated anti-chicken IgG, Cy2-conjugated goat anti-mouse IgG or Cy2-conjugated donkey anti-goat IgG). Negative control sections were incubated with isotype-specific non-immune IgG or normal goat serum. Confocal fluorescence images were captured with a laser scanning. Quantitative evaluation of single positive cells in the SVZ and hippocampus areas was carried out on ten cryostat sections at X40 magnification for each mouse using the Image Pro-Plus software (version 1.43 u).

### Statistical analysis

The limma empirical Bayesian implementation of the t-test [59] was used to compare experimental data derived from miRNA and mRNA molecular and immunohistochemistry analyses between G93A-SOD1 and control mice. A Bonferroni correction was applied to control for false discovery rate; *p* < 0.05 was considered significant. Pearson correlation coefficients (r) were determined to identify relationships between miRNAs and target mRNA expression levels [60] in different brain regions of 18 week-old G93A-SOD1 mice. The R statistical environment (www.r-project.org) was used for statistical analyses.

## Additional files

Additional file 1: Figure S1. Expression levels of neural- and cell cycle-related miRNAs in G93A-SOD1 spinal cord as disease progresses. RT-PCR analysis of neural-specific and cell cycle-related miRNAs in total RNA extracted from whole spinal cord of G93A-SOD1 and Wt-SOD1 mice, at postnatal week 18 (ten mice per group). Each point represents a single spinal cord. Relative expression data are presented as mean ± SD. **p* < 0.05; ***p* < 0.01; limma moderated t-test. Additional file 2: Figure S2. Expression levels of glial-related miRNAs in spinal cord regions. RT-PCR analysis of miR-125b and miR-219, implicated in astrocyte and oligodendrocyte functional regulation, in cervical, thoracic and lumbar spinal cord regions. Total RNA was extracted from cervical, thoracic and lumbar spinal cord of G93A-SOD1 and Wt-SOD1 mice, at postnatal week 18 (three mice per group). Relative expression data are presented as mean ± SD; limma moderated t-test.

## Abbreviations

(ALS): Amyotrophic lateral sclerosis
(Ccnd2): Cyclin D2
(Dlx2): Distal-less homeobox 2
(FoxJ3): Forkhead box protein J3
(GFAP): Glial fibrillary acidic protein
(Hes1): Hairy and enhancer of split
(Jag1): Jagged
(Mknk2): MAP kinase interacting serine/threonine kinase 2
(miRNAs): microRNAs
(NSPCs): Neural stem progenitor cells
(Nr2e1): Nuclear receptor subfamily
(Pten): Phosphatase and tensin homolog
(SRY): Sex determining region Y
(Sox6): Box 6
(SRY): Sex determining region Y
(Sox9): Box 9
(STAT3): Signal transducer and activator of transcription 3
(Smurf1): Specific E3 ubiquitin protein ligase 1
(SGZ): Subgranular zone
(SVZ): Subventricular zone
(Socs1): Suppressor of cytokine signalling 1
