# Supplementary material for: Up-regulation of neural and cell cycle-related microRNAs in brain of amyotrophic lateral sclerosis mice at late disease stage
Source: Mol Brain. 2015 Jan 28;8:5. doi: 10.1186/s13041-015-0095-0 (PMC4318136; doi:10.1186/s13041-015-0095-0)
Supplement: Additional file 1: Figure S1. — Expression levels of neural- and cell cycle-related miRNAs in G93A-SOD1 spinal cord as disease progresses. RT-PCR analysis of neural-specific and cell cycle-related miRNAs in total RNA extracted from whole spinal cord of G93A-SOD1 and Wt-SOD1 mice, at postnatal week 18 (ten mice per group). Each point represents a single spinal cord. Relative expression data are presented as mean ± SD. *p < 0.05; **p < 0.01; limma moderated t-test. [file 13041_2015_95_MOESM1_ESM.doc]

**Additional File: Figure 1**


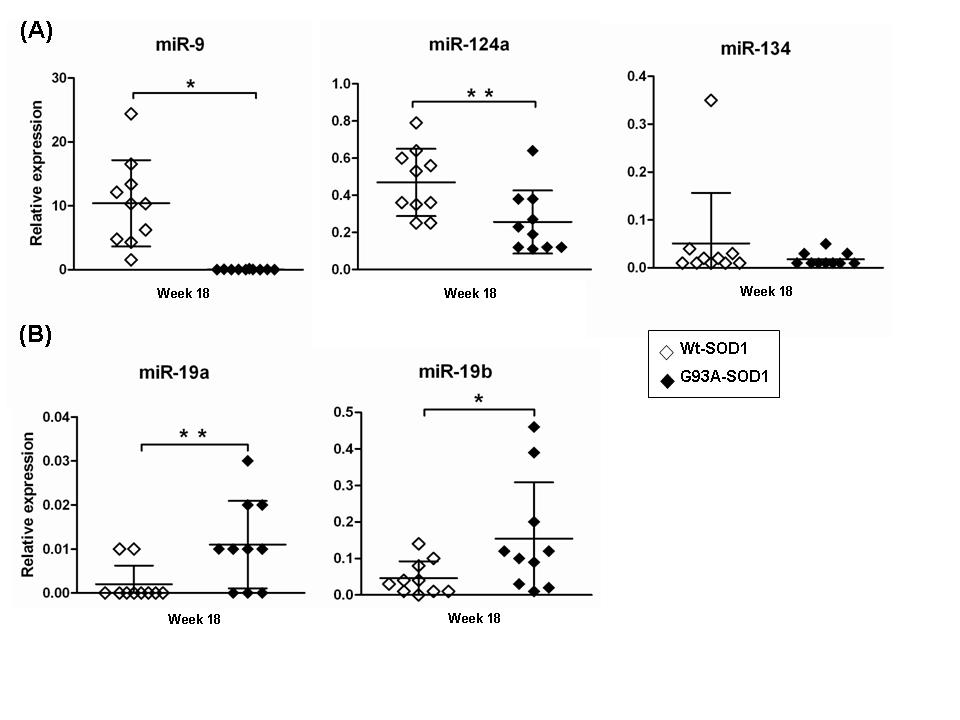


**Additional Figure 1.** Expression levels of neural and cell-cycle related miRNAs were altered in G93A-SOD1 mouse spinal cord as disease progresses. RT-PCR analysis of neural-specific (A) and cell-cycle-related (B) miRNAs in total RNA extracted from whole spinal cord of G93A-SOD1 and Wt-SOD1 mice, at postnatal week 18 (ten mice per group). Each point represents a single spinal cord. Relative expression data are presented as mean ± SD. *p<0.05; **p < 0.01; Limma moderated t-test.
