# Supplementary material for: Up-regulation of neural and cell cycle-related microRNAs in brain of amyotrophic lateral sclerosis mice at late disease stage
Source: Mol Brain. 2015 Jan 28;8:5. doi: 10.1186/s13041-015-0095-0 (PMC4318136; doi:10.1186/s13041-015-0095-0)
Supplement: Additional file 2: Figure S2. — Expression levels of glial-related miRNAs in spinal cord regions. RT-PCR analysis of miR-125b and miR-219, implicated in astrocyte and oligodendrocyte functional regulation, in cervical, thoracic and lumbar spinal cord regions. Total RNA was extracted from cervical, thoracic and lumbar spinal cord of G93A-SOD1 and Wt-SOD1 mice, at postnatal week 18 (three mice per group). Relative expression data are presented as mean ± SD; limma moderated t-test. [file 13041_2015_95_MOESM2_ESM.doc]

**Additional File: Figure 2**

**
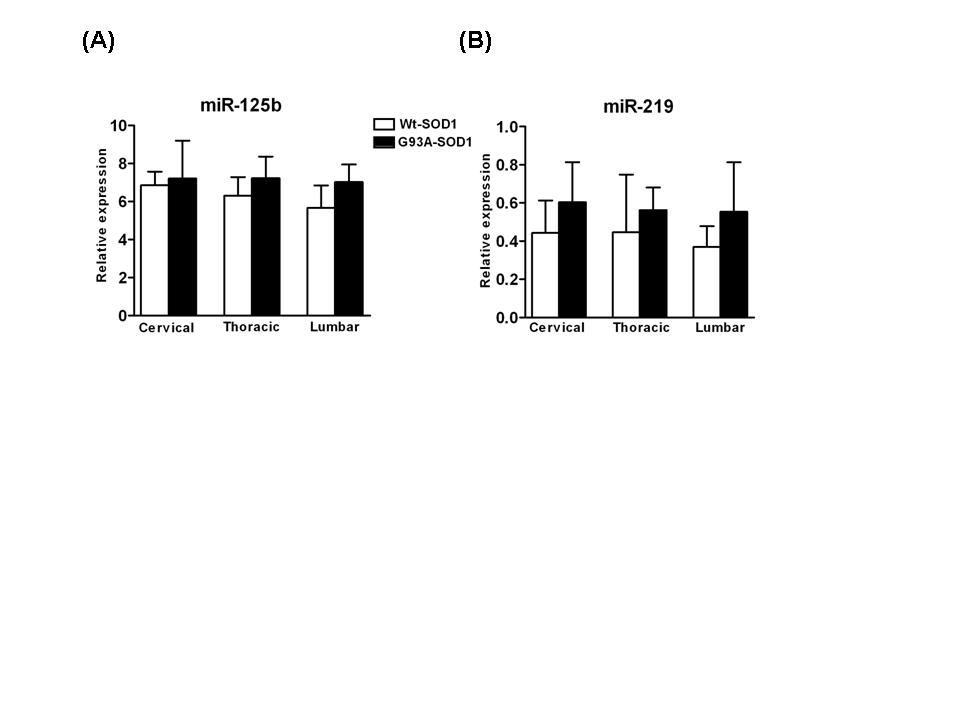
**

**Additional Figure 2.** Expression levels of glial-related miRNAs in spinal cord regions. RT-PCR analysis of miR-125b (A) and miR-219 (B), implicated in astrocyte and oligodendrocyte functional regulation, in cervical, thoracic and lumbar spinal cord regions. Total RNA was extracted from cervical, thoracic and lumbar spinal cord of G93A-SOD1 and Wt-SOD1 mice, at postnatal week 18 (three mice per group). Relative expression data are presented as mean ± SD. Limma moderated t-test.
